# Handbook of Practical Medicine

**Published:** 1887-01

**Authors:** 


HANDBOOK OF PRACTICAL MEDICINE. Vol. I. Diseases of the Circulatory and Respiratory Apparatus. Vol. II. Diseases of the Digestive, Urinary and Sexual Apparatus. Vol. III. Diseases of the Nerves, Muscles and Skin. By Hermann Eichorst, Professor of Special Pathology and Therapeutics and Director of the University Medical Clinic in Zurich, being Vols. III., VI. and X. of Wood's Library of Standard Medical Authors, 1886 (consisting of 12 Vols., price, $15.00). Sold only by subscription. William Wood and Company, New York.

Vol. I. is divided into two sections. Section first treating of diseases of the pericardium, heart muscle, endocardium, neuroses of the heart and diseases of the aorta. Section second is devoted to diseases of the nasal cavities, larynx, trachea, bronchi, lungs, pleura, pulmonary artery, mediastinum.

Vol. II. on diseases of the buccal cavity and pharynx, œsophagus, stomach, intestines, liver, pancreas, peritoneum, changes in the urine, diseases of the renal parenchyma, renal pelvis and ureters, bladder and male sexual apparatus.

Vol. III. discusses diseases of the peripheral nerves, spinal cord, medulla oblongata, brain, sympathetic system, diseases of the muscles and diseases of the skin, embracing inflammations, anomalies of secretion, hypertrophy, atrophy, neuroses and parasites. In the discussion of each disease the etiology, anatomical changes, symptoms, diagnosis, prognosis and treatment are given, and the reader is at once impressed with the eminently practical tone that everywhere characterizes the work. The style of the writer is clear and vigorous without being dogmatic, each subject being handled in a masterly manner. The illustrations are abundant, and those representing changes in the urine and diseased conditions of the nervous system merit special mention. These volumes form a valuable acquisition to the library, and the publishers deserve the thanks of the profession for their enterprise in securing the works of such famous authors.

